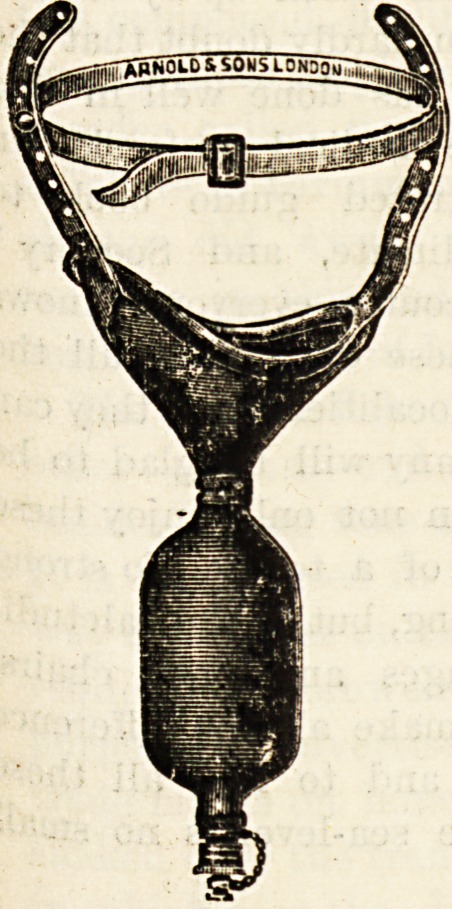# New Appliances and Things Medical

**Published:** 1900-11-17

**Authors:** 


### NEW APPLIANCES AND THINGS MEDICAL.

[We shall be glad to receive, at our Office, 28 & 29 Southampton Street, Strand, London, W.C., from the manufacturers, specimens of all new preparations and appliances which may be brought out from time to time.]

### DUKE'S INFANT URINAL.

(Arnold and Sons, 26 West Smithfield, London, E.C.)

We have examined a specimen of this urinal, but have not at present been able to put the appliance to practical test. The inventor, Dr. Alexander Duke, has designed the urinal as a substitute for the towelette. It is made of india-rubber, and consists of a double chamber, one of which is to be applied to the perinæum covering the anus and the external genitals the second chamber, which is connected to the first by a narrow neck, retains the urine perfectly safely. In no position can the contents be upset. They are emptied when necessary by opening the tap at the lower end. The saving of time and trouble by the substitution of this ingenious appliance on the face of it looks very attractive, and we regret that we have not been able to keep the sample sent us and submit it to practical trial. There is one use, however, to which this urinal may be applied with the greatest advantage—namely, the collection of samples of urine for clinical test purposes. Examination of infants' urine has been considerably neglected in the past, for the very reason that it is so difficult to obtain. Many practitioners would be surprised if they only knew how frequently sugar is present in the urine of over-fed rickety children, and how often the urea exists in almost pathological percentages. Routine examination, which is now made possible by this simple contrivance, will do much to further our knowledge with regard to the usual and unusual conidtions of the urine in infants.

### BYNO-HÆMOGLOBIN.

(Allen and Hanburys, Limited, Plough Court, Lombard Street, London, E.C.)

This new preparation of organised iron has, in our opinion, many obvious advantages over the inorganic salts of this base which are constantly employed in cases of anæmia, whether symptomic or of the specific form of chlorosis. Whatever be the therapeutic action of the inorganic preparations of iron, it is practically certain that they do not fulfil the objects for which they are usually prescribed, namely, to enter into direct combination with the organic elements of the blood. It is possible, however, that iron in the form of hæmoglobin may be assimilated and built up into living protoplasm, when supplied in an unchanged form, such as byno-hæmoglobin. In this preparation hæmoglobin is dissolved in bynin (liquid malt) in the proportion of one in eight. The hæmoglobin is prepared from pure blood by a special cold process whereby the iron is not discharged from its organic combination. As far as our experience goes this new form of iron and maltine gives results which fulfil the expectations which one would naturally form on theoretical grounds from the constitution of the ingredients.

### MARROWLENE.

(Marrowlene Factory, Blanchardstown, Dublin.)

Marrowlene is a specially prepared fat, which is offered as a substitute for butter, lard, or dripping. It should prove a useful and economical preparation for ordinary domestic use. It is supplied in solid blocks of two pounds in weight. It differs from margarine in consistence and keeping properties, and, compared with it, it contains a much smaller percentage of water. It seems to answer capitally for making pastry, cakes, puddings, &c., and to be free from all objectionable preservatives.

### TABLETS OF FERRICHTHOL AND ICHTHYOL CALCII.

In reference to the notice of these preparations which appeared in our issue of last week, we desire to point out that they ought to be described as "tablets" not "tabloids."

## Figures and Tables

**Figure f1:**